# Network model predicts that CatSper is the main Ca^2+^ channel in the regulation of sea urchin sperm motility

**DOI:** 10.1038/s41598-017-03857-9

**Published:** 2017-06-26

**Authors:** Jesús Espinal-Enríquez, Daniel Alejandro Priego-Espinosa, Alberto Darszon, Carmen Beltrán, Gustavo Martínez-Mekler

**Affiliations:** 1 Computational Genomics Division, National Institute of Genomic Medicine (INMEGEN), 14610 Mexico City, Mexico; 2 Centro de Ciencias de la Complejidad, Universidad Nacional Autónoma de México (UNAM), Mexico City, 04510 Mexico; 3 Instituto de Ciencias Físicas, Universidad Nacional Autónoma de México, Cuernavaca, Morelos 62210 Mexico; 4 Instituto de Biotecnología, Universidad Nacional Autónoma de México, Cuernavaca, Morelos 62210 Mexico

## Abstract

Spermatozoa sea urchin swimming behaviour is regulated by small peptides from the egg outer envelope. Speract, such a peptide, after binding to its receptor in *Strongylocentrotus purpuratus* sperm flagella, triggers a signaling pathway that culminates with a train of intracellular calcium oscillations, correlated with changes in sperm swimming pattern. This pathway has been widely studied but not fully characterized. Recent work on *Arbacia punctulata* sea urchin spermatozoa has documented the presence of the Ca^2+^ CatSper channel in their flagella and its involvement in chemotaxis. However, if other calcium channels participate in chemotaxis remains unclear. Here, based on an experimentally-backed logical network model, we conclude that CatSper is fundamental in the *S. purpuratus* speract-activated sea urchin sperm signaling cascade, although other Ca^2+^ channels could still be relevant. We also present for the first time experimental corroboration of its active presence in *S. purpuratus* sperm flagella. We argue, prompted by *in silico* knock-out calculations, that CatSper is the main generator of calcium oscillations in the signaling pathway and that other calcium channels, if present, have a complementary role. The approach adopted here allows us to unveil processes, which are hard to detect exclusively by experimental procedures.

## Introduction

In their search for the egg, *S. purpuratus* and *Lytechinus pictus* sea urchin spermatozoa respond to speract, a decapeptide present in the eggs’ external layer that diffuses into the sea. When speract binds to its receptor, which is located in sperm flagellar membrane, a signaling cascade initiates. This biochemical pathway is finely regulated by changes in membrane permeability to certain ions. The ensuing speract-activated signaling pathway (SASP) produces a train of intracellular *Ca* ^2+^ concentration ([*Ca* ^2+^]_*i*_) oscillations. These [*Ca* ^2+^]_*i*_ fluctuations are correlated with changes in the sperm swimming behaviour. Under two dimensional experimental conditions, sea urchin spermatozoa swim describing circles close to surfaces. When flagella detect increases in *Ca* ^2+^ influx rate, their beating becomes asymmetric and the spermatozoon undergoes pronounced turns that are then followed by straighter swimming^1, 2^.

## Preliminaries

### Previous proposal for the *S. purpuratus* [*Ca*^2+^]_*i*_ signaling pathway

After speract binding, the speract-receptor complex activates a membrane guanylate cyclase (GC) which in turn produces cGMP, this second messenger opens a cGMP-dependent *K* ^+^ channel (KCNG) that hyperpolarizes the membrane potential. Hyperpolarization influences several processes: i) it stimulates a *K* ^+^/*Na* ^+^/*Ca* ^2+^ exchanger (NCKX)^3–7^ that decreases [*Ca* ^2+^]_*i*_, ii) activates a *Na* ^+^/*H* ^+^ exchanger (NHE)^5, 6^ which increases intracellular pH (*pH*_*i*_), iii) diminishes inactivation of high and low voltage-dependent calcium channels (HVA and LVA, respectively) and iv) stimulates a voltage-activated cAMP-dependent *Na* ^+^ channel (HCN) that depolarizes^6^. The pHi elevation stimulates a soluble adenylyl cyclase (sAC)^7^ which synthesizes cAMP that in turn opens the previously mentioned HCN channel, as well as a cAMP-dependent *Ca* ^2+^ channel (cAMPCC) that repolarizes the membrane. Via this chain of events, the cAMPCC acquires a pH dependence. Cyclic nucleotides levels are strongly controlled by phosphodiesterases (PDE) which degrade them. The cAMP dependence of this channel may be indirect. Repolarization allows the opening of LVA and HVA *Ca* ^2+^ channels; these channels have been thought to be responsible for the [*Ca* ^2+^]_*i*_ fluctuations^6, 8–15^. [*Ca* ^2+^]_*i*_ increases may open calcium-dependent *K* ^+^ channels (CaKC) and chloride channels (CaCC). These last two channels can restart the pathway through a new hyperpolarization^10, 16, 17^. Calcium extrusion is carried out by the calcium pumps (CaP), and the above mentioned NCKX. The cyclic alternation of hyperpolarization and repolarization generates the train of oscillations in [*Ca* ^2+^]_*i*_ that govern the sea urchin sperm swimming pattern^16^. Despite that this SASP has been widely studied, many questions remain regarding participating elements and their relations, mainly due to experimental limitations.

## SASP network model

In the logical signaling network corresponding to the SASP, first introduced in ref. 16, nodes represent the aforementioned components: ion channel activities, intracellular ion and molecular concentrations and the membrane potential, amongst others, and links are indicative of functional dependencies between nodes. In the model, a discrete-time-step dynamics is considered for the operation of the network, where nodes can take up to three values from the set 0, 1, 2 which are updated, according to a set of regulatory rules, at each time iteration. This is a generalization of the Boolean network approach that has proven to be revealing for genetic, proteomic, metabolic and transcriptional networks, amongst others^18–23^. The state of the network consists of a set of *N* discrete variables *σ*_1_, *σ*_2_, …, *σ*_*n*_. Most of the variables take on two values, namely, 0 for closed/off and 1 for on/open states. However, an accurate description of the dynamical processes in the network required four nodes to be represented by three-state variables: the membrane potential (hyperpolarized 0, resting 1, and depolarized 2); the LVA and HVA *Ca* ^2+^ channels (inactive 0, closed 1, and open 2); and the intracellular calcium concentration ([*Ca* ^2+^]_*i*_) (basal 0, tonic 1 and supratonic 2). The state of each node *σ*_*n*_ is determined by its set of regulators, i.e. the nodes functionally linked to it. Let us denote as ${\sigma }_{{n}_{1}}$, ${\sigma }_{{n}_{2}}$, …, ${\sigma }_{{n}_{k}}$ the *k* regulators of *σ*_*n*_. Then, at each time step the value of *σ*_*n*_ is given by

1 $${\sigma }_{n}(t+\mathrm{1)}={F}_{n}({\sigma }_{{n}_{1}},{\sigma }_{{n}_{2}},\ldots ,{\sigma }_{{n}_{k}}),$$

where *Fn* is the regulatory function specific to that node. For the construction of these regulatory functions (shown in Supplementary Dataset S1) we have made use of extensive biological knowledge, available to us in the literature and from our own laboratory. With this model we can observe *in silico* the effect of altering certain elements relevant to the pathway. Hereafter we shall refer to this setup as Model-I.

## SASP physiological considerations

Experimental observations have shown that in *S. purpuratus* sperm populations^6^ and now in single cells^24^, speract first increases pHi and then [*Ca* ^2+^]_*i*_, suggesting that the sperm specific Na^+^/H^+^ exchanger (sNHE)^25^ may plays a role in the first stages of the pathway. Though the fine regulation of *pH*_*i*_ via sNHE is not yet fully understood, the establishment of a direct link with [*Ca* ^2+^]_*i*_ fluctuations is appealing. A strong candidate for this link is the calcium channel CatSper, since in mammals it has been shown to be sperm specific^26–28^, activated by alkalization, mildly voltage-sensitive and essential for fertilization^26, 28, 29^. Additionally, the sequences that codify this channel have been found in *S. purpuratus* genome^30^. The participation of a *pH*_*i*_ dependent *Ca* ^2+^ channel in *S. purpuratus* sperm physiology was conjectured in ref. 31 and recently CatSper was identified in the *A. punctulata* sperm flagella^32^. What remains to be shown is whether or not it is the principal generator of oscillations in the signaling pathway of *S. purpuratus* and if other calcium channels are accessory. If this were the case, CatSper would be the main controller of the [*Ca* ^2+^]_*i*_ oscillations and consequently of the sperm swimming behaviour.

## Model-related considerations

Our initial logical network recovered several experimental observations and produced predictions which were later experimentally validated^16^. In that study, it was shown that an *in silico* blockage of the CaKC increased the time between subsequent calcium peaks as well as the average [*Ca* ^2+^]_*i*_. However, when experiments with Iberiotoxin, a specific blocker of the CaKC channel^33^, were carried out, it was observed that though the time between calcium peaks of the speract-dependent [*Ca* ^2+^]_*i*_ oscillations increased, the average [*Ca* ^2+^]_*i*_ diminished. As a way out of this disagreement, the existence of other ionic channels in the pathway not previously taken into account was suggested.

In a more recent paper^34^ we studied with our model the effect of niflumic acid (NFA) on the SASP by considering its action on the permeability of CaCC, HCN and CaKC. Though with slight differences, NFA affects these three channels in the *μ*M range: CaCC^35–40^, CaKC^41, 42^, and HCN^40, 43–45^.

Two scenarios emerged depending on the experimental controversy of whether the action of NFA on CaKC is inhibitory or activating. Taking into account the recently accumulated evidence regarding the activation of CaKCs by NFA, our model required the participation of more NFA-sensitive ionic channels in the SASP in order to recover the experimental results performed by ref. 17. In that paper we suggested that CatSper might well be such a channel.

## Outline

In this paper we address the necessity of an additional *pH*_*i*_-dependent *Ca* ^2+^ channel in the *Ca* ^2+^ signaling pathway. Prompted by the above, we added the *pH*_*i*_, voltage dependent CatSper channel to the network of ref. 16. With this enhanced network, labeled as Model-II we analyzed the degree of participation in the SASP of CatSper with respect to the other main *Ca* ^2+^ channels previously considered (HVA and LVA), by performing *in silico* knock-outs in our model network. The outcome that CatSper channel plays a leading role with regard to the amplitude of the *Ca* ^2+^ fluctuations, lead us to a Model-III signaling pathway, where the only remaining *Ca* ^2+^ channel is CatSper. With the three models at hand, we proceeded with a comparative study of their dynamics that lead us to the prediction that the CatSper channel participates in the *S. purpuratus* SASP and appears to be the principal generator of [*Ca* ^2+^]_*i*_ oscillations, while the other calcium channels considered in this study are probably secondary. Furthermore, our results prompted us to the design of pharmacological protocols to examine whether CatSper participates in the [*Ca* ^2+^]_*i*_ response induced by speract in *S. purpuratus* sperm. We confirmed by proteomics the presence of all CatSper subunits but Catsper3 (as shown in Table 1). There after, we tested known channel blockers of this channel like Mibefradil and NNC-055–396 (NNC)^32, 46, 47^. These inhibitors diminished significantly the increase in [*Ca* ^2+^]_*i*_ induced by speract, reinforcing the proposal that CatSper plays a role in this response. Analogous results were recently found for *A. punctulata* spermatozoa stimulated by resact^32^.

**Table 1:** Different cation channel sperm-associated proteins (CatSper) family members identified by proteomic analysis, including sequence ID: ref numbers, molecular weights (MW; KDa) and peptide amino acid sequences.

Name	Sequence ID: ref	MW (KDa)	Unique Peptide sequence
Catsper 1	XP_011664201.1	55	(R)SAHAYQAHDINKHEDEEEEELEVRR(N)
			(K)TIDDYYDEEDYSTSEKK(Y)
			(K)YLSHFLQLLASIEHNGHILR(N)
			(R)NQQGTLDKVVLLVQDTVDDA(-)
Catsper 2	XP_011679404.1	60	(K)NKVEIDDTPR(L)
Catsper 4	XP_011682209.1	36	(K)NKVEIDDTPR(L)
			(R)LFTFDPNAIQR(G)
Catsper *β*	XP_011679115.1	52	(K)TAVGQEIVGSGMR(L)
			(R)LLITNMR(S)
			(R)SLSDGTLVAMATSQEPSLHANMR(T)
			(R)TPDIAENVIDLLLK(E)
			(K)GVSSILISIPSASLR(C)
			(R)LHFIYDPPVTSQEFLYGSPK(N)
			(R)LGISVPLTNNIYNADPSQPR(L)
			(R)GKDDEVLAAPYILTIK(E)
			(K)EVNDREDFVIEAADVVYmSEIGK(V)
			(R)LTVIDGFSYcKLEDEVQVFVDR(A)
CatSper *δ*	XP_011664202.1	36	(K)AEPEEDHEQGDNSDD(-)
Catsper *γ*	XP_011679048.1—	82	(R)YFYQYLFQNK(N)
			(K)NLQSGIHIDPDAYYLQPFGNTPEDTK(T)
			(K)VVDFTTGEASLFEGLYTLTVL(G)
			(G)VSHSSEEDIVLFTPEEQQR(Y)
			(K)GGSPCSDVPPSK(L)
			(R)EGIAHRPIGLSTTSTVIER(L)
			(R)SVSSNILDR(S)
			(R)YNNLDISGSR(I)

## Results

### The model shows that the CatSper channel could participate in shaping the [*Ca*^2+^]_*i*_ oscillation pattern in the SASP

**Figure 1:**
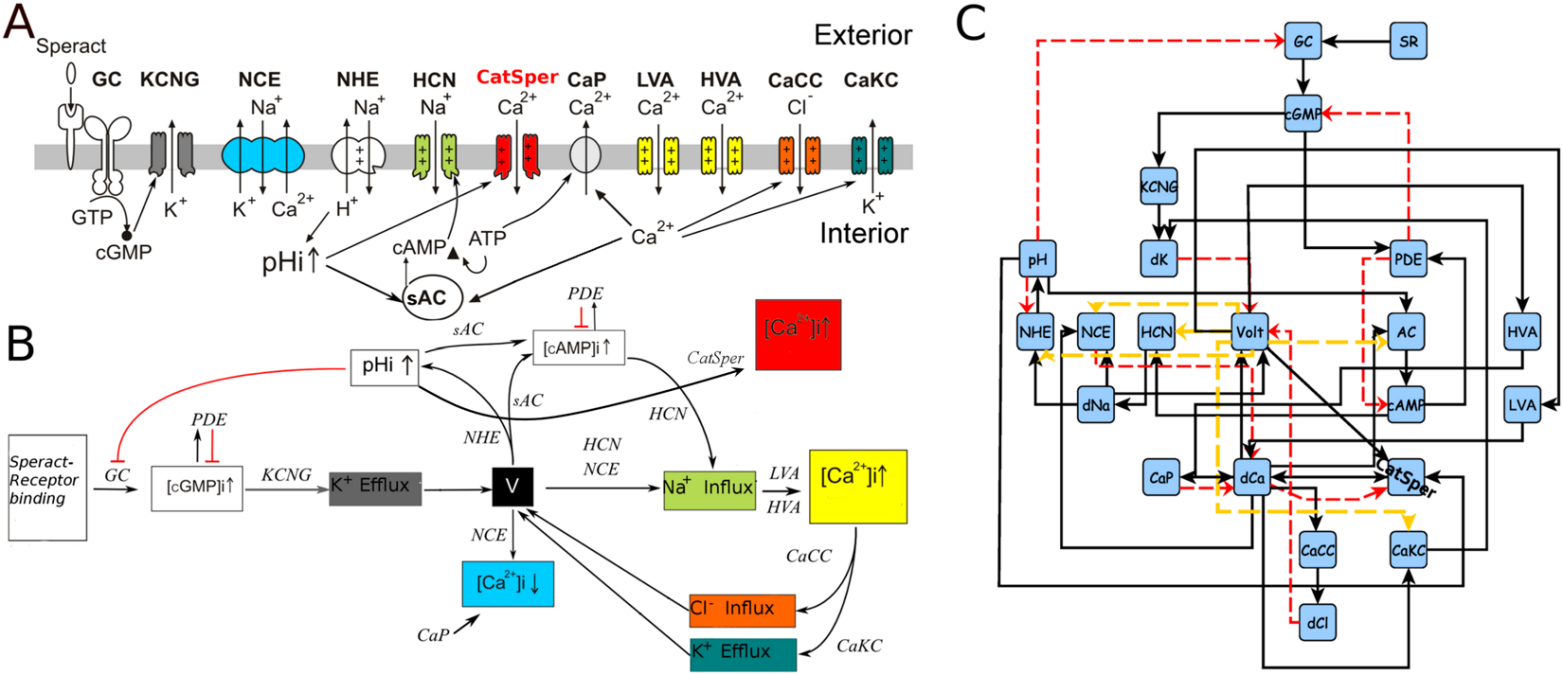
Model-II signaling Pathway and Logical Network of the Speract-Activated Signaling Pathway (SASP). (**A**) Scheme of the signaling pathway triggered by speract in *S. purpuratus* sperm flagellum. The SASP starts with the binding of speract to its receptor and after several steps, which include changes in membrane potential, oscillations in [*Ca* ^2+^]_*i*_ are attained. (**B**) Biochemical events related to (**A**). (**C**) Network model of the signaling pathway. Each node on the network represents an element of the pathway. Black arrows indicate activation; red lines indicate inhibition and the dashed yellow arrows can both activate and inhibit depending on the value of voltage V. Voltage can have a hyperpolarized state, represented with a value of 0; resting potential, 1 and a depolarized state, which is represented with a value of 2.

Figure 1 shows Model-II SASP and corresponding logical network (regulatory functions for Model-II can be found in Supplementary Dataset S2). In order to obtain a description of the [*Ca* ^2+^]_*i*_ closer to the experimental observations, which are single cell averaged out measurements along the flagellum, we average the value of [*Ca* ^2+^]_*i*_, over 100,000 initial conditions, as in refs 16, 34. Besides providing a variable more accurate for experimental comparisons, this averaged value, as will be seen further on, contributes to a better understanding of the dynamics.

Our finding is that the contribution of the CatSper channel to the *Ca* ^2+^ is able to produce oscillations and overshadows the other two *Ca* ^2+^ channels (HVA and LVA) previously considered in the SASP. The presence of this channel is further substantiated with the result that, with its addition, aforementioned shortcomings encountered in Model-I can be overcome.

### CatSper plays a central role in the SASP

**Figure 2:**
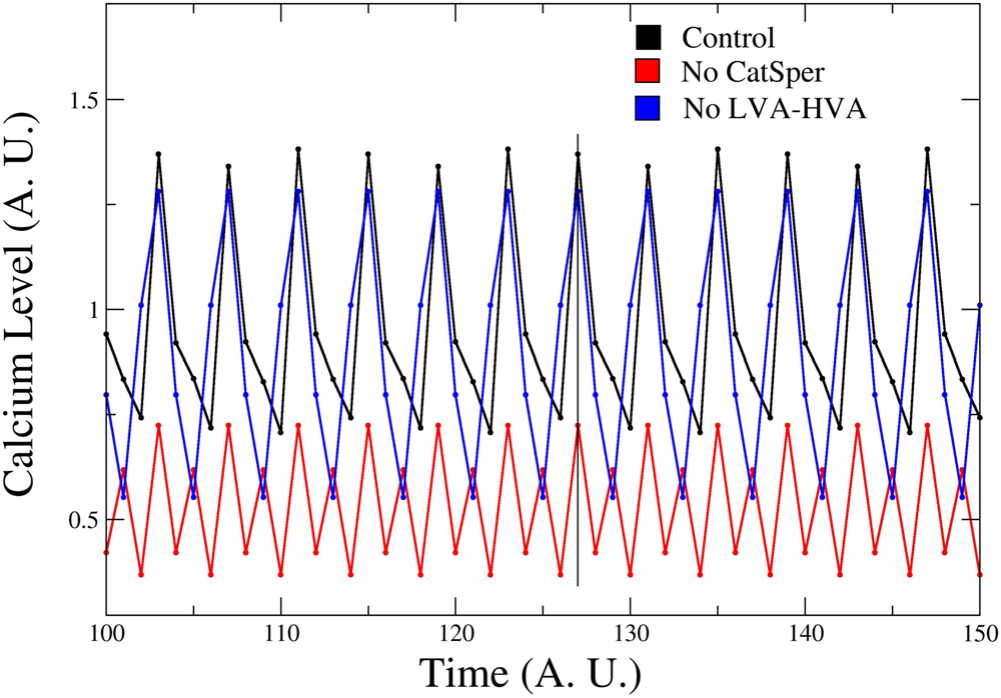
Time series of the network dynamics of the [*Ca* ^2+^]_*i*_ under the blockage of the different *Ca* ^2+^ channels present in the sea urchin sperm flagellum: HVA-LVA and CatSper. The [*Ca* ^2+^]_*i*_ curve with all nodes present (WT) is depicted in black. Red line corresponds to the [*Ca* ^2+^]_*i*_ dynamics after blockage of the CatSper channel. The blue line represents the [*Ca* ^2+^]_*i*_ nodes of the network dynamics without both HVA and LVA channels. The X axis units are number of iterations of the network dynamics. The Y axis units are arbitrary, indicative of relative [*Ca* ^2+^]_*i*_ node values.

In Fig. 2 we show in black the values of *Ca* ^2+^ for Model-II averaged over 100,000 uniformly distributed random initial conditions determined from equation (1) as explained in the materials and methods section, using the regulatory functions corresponding to Fig. 1. We also plot in red the case in which the CatSper node has been blocked, keeping the HVA and LVA nodes active, and in blue the case in which both HVA and LVA channels have been blocked leaving CatSper present. The blockage of CatSper can be observed to produce a drastic decrease on the *Ca* ^2+^ levels in comparison with the joint blockages of HVA and LVA, placing it as the main source of *Ca* ^2+^ intake. Additionally, the oscillatory temporal behaviour of the *Ca* ^2+^ fluctuations is modified by the appearance of an additional small peak within the wild type period.

CatSper hence also plays a role in frequency oscillation control in our model. On the other hand, the joint blockage of HVA and LVA channels has no effect on the period of oscillation. However, for this case there is a one step phase difference with regard to the wild type noticeable in the minima positions, which causes the loss of the original activation-relaxation asymmetry. Hence, though CatSper is the dominant channel for the network regulation of the *Ca* ^2+^ fluctuations, the other channels may still play a role.

**Figure 3:**
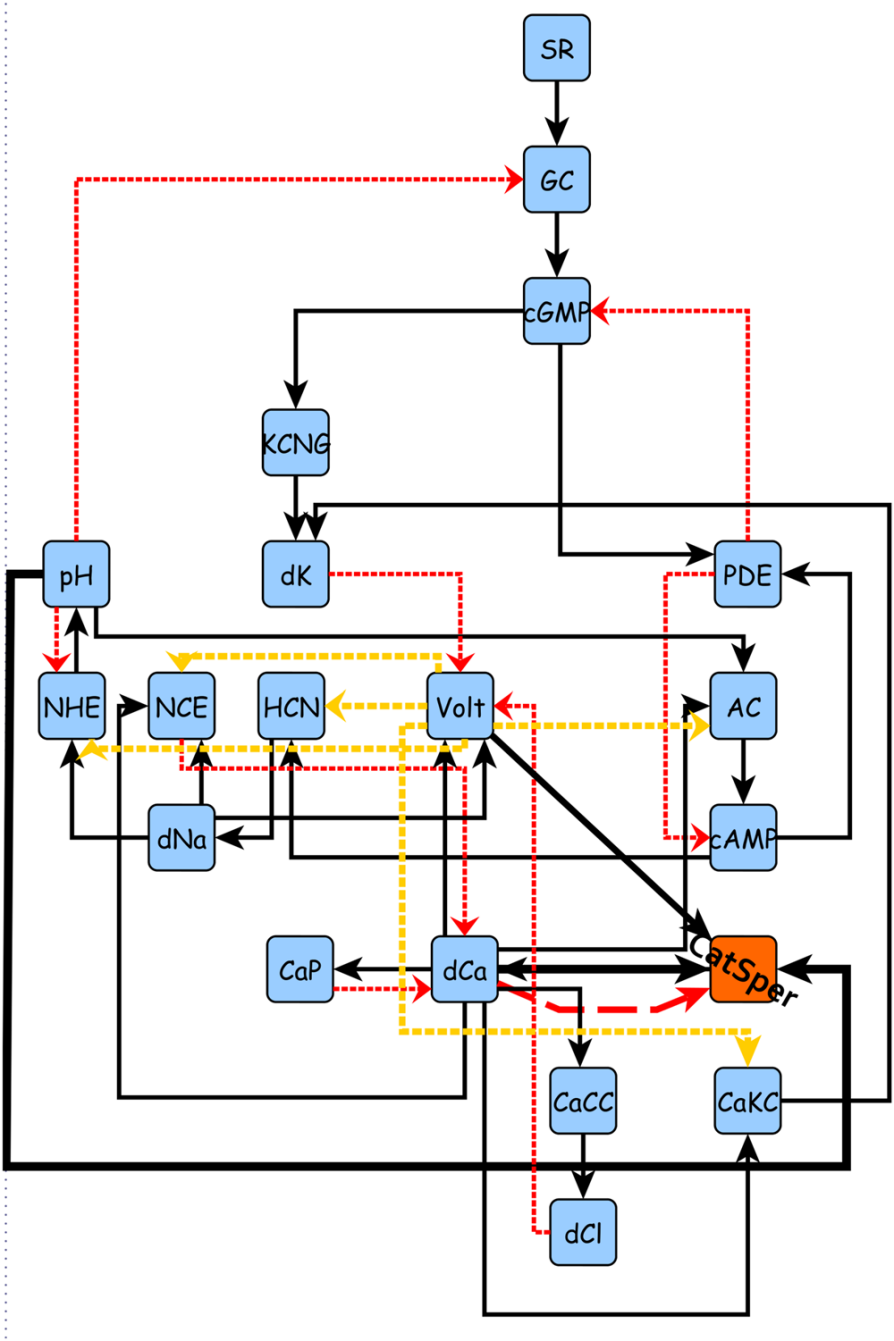
Network representation of the signaling pathway with CatSper channel as the only source of intracellular *Ca* ^2+^ concentration. Notice the absence of the LVA and HVA channels previously depicted in Fig. 1. Also note the new direct link between pH, voltage and *Ca* ^2+^ node with the CatSper channel, highlighted with bold arrows. This network constitutes Model-III.

Keeping the above in mind from now on we will present a comparative study amongst Model-I, Model-II and Model-III for which CatSper is the only *Ca* ^2+^ channel. The network for Model-III is shown in Fig. 3 and the regulatory functions are defined in Supplementary Dataset S3.

### On PDE dependence

**Figure 4:**
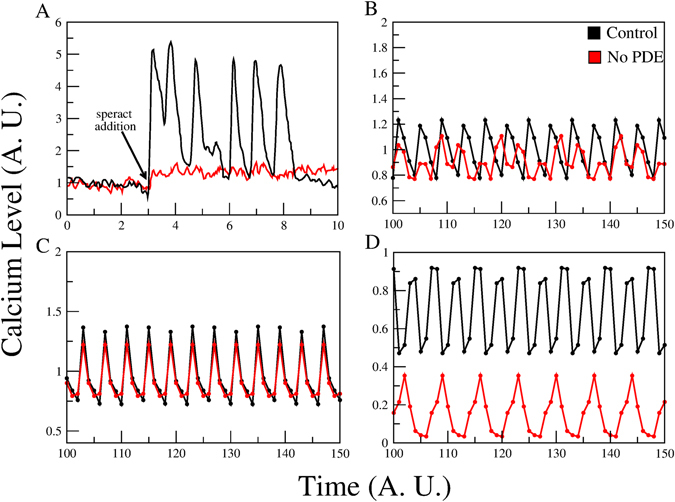
[*Ca* ^2+^]_*i*_ dynamics of the network model with and without the PDE node. Panel A shows a typical experimental measurement of [*Ca* ^2+^]_*i*_ florescence after exposure to speract. The control curve is depicted in black; the red curve represents the calcium level with speract + IBMX, a known PDE blocker. Note the considerable decrease in the *Ca* ^2+^ induced by PDE blockage. Panels B,C and D are the time series of the *Ca* ^2+^ level for the control curve (black) and under deletion of the PDE node (red) for Models I, II and III respectively. In the time series of panel B, the average calcium level is slightly lower and its oscillations smaller under PDE deletion. Model-II (panel C) remarkably displays only small changes. However, for Model-III (panel D), the calcium level under PDE-blockage is drastically reduced.

It was reported that exposure to 3-isobutyl-1-methylxanthine (IBMX), a nonspecific PDE blocker, diminishes speract-dependent *Ca* ^2+^ oscillations^17^. In Fig. 4 we present a comparison between the experiment and the results from steady-state *in silico* blockage of PDE in the three models. Panel A shows experimental results related to IBMX exposure. In panel B, Model-I is shown to produce a small decrease in the average values of *Ca* ^2+^ with respect to the wild type and the temporal behaviour becomes irregular displaying smaller oscillations. For panel C, where Model-II is considered, the participation of HVA and LVA, together with a contribution from CatSper produces, under PDE suppression, oscillations that almost overlap the WT behaviour. While in panel D, Model-III qualitatively reproduces the experimental results of a considerable decrease in *Ca* ^2+^ level.

The results for Model-III (CatSper only, panel 4D) may be explained by considering that IBMX is a nonspecific phosphodiesterase inhibitor that allows accumulation of both cGMP and cAMP in all cell compartments. In a regular speract response cGMP first elevates in the flagella and with a delay cAMP then goes up^9, 48^. The delay between the cGMP elevation and that of cAMP allows the KCNG channel to first open and hyperpolarize so that when cAMP begins to increase HCN can open and depolarize. Sperm population speract responses in the presence of IBMX have shown that membrane potential remains hyperpolarized indicating that cGMP activation of KCNG prevails over the cAMP induced depolarization caused by SpHCN^49^. IBMX wrecks the kinetic and spatial coordination between these two channels and [*Ca* ^2+^]_*i*_ increases but cannot decrease or oscillate. Since the CatSper channel requires a depolarization to activate even though a high hyperpolarization elevates pH_*i*_, CatSper will not open and a lower *Ca* ^2+^ level will result.

Since the CatSper channel is a depolarization-activated *Ca* ^2+^ channel, a high hyperpolarization decreases the normal activity of CatSper, which in turn produces a lower *Ca* ^2+^ level.

For the case of Model-I (panel B) with HVA, LVA and a cAMP-dependent *Ca* ^2+^ channel; concomitant with the cGMP-dependent steps, which lead to a *Ca* ^2+^ decrease, the absence of PDE also increases cAMP activity, which in turn stimulates a cAMP-modulated *Ca* ^2+^ channel. So, in this case, we have a double *Ca* ^2+^ regulation via cyclic nucleotides: an inhibitory (hyperpolarization-mediated) and an activator (cAMP-modulated *Ca* ^2+^ channel opening). While for Model-II (panel C), the interplay between these opposite processes produces a higher *Ca* ^2+^ level compared to the Model-III. The overall conclusion is that agreement with our results supports a dominant role displayed by CatSper with regard to the other *Ca* ^2+^ channels: HVA and LVA.

### On *pH*_*i*_ dependence

**Figure 5:**
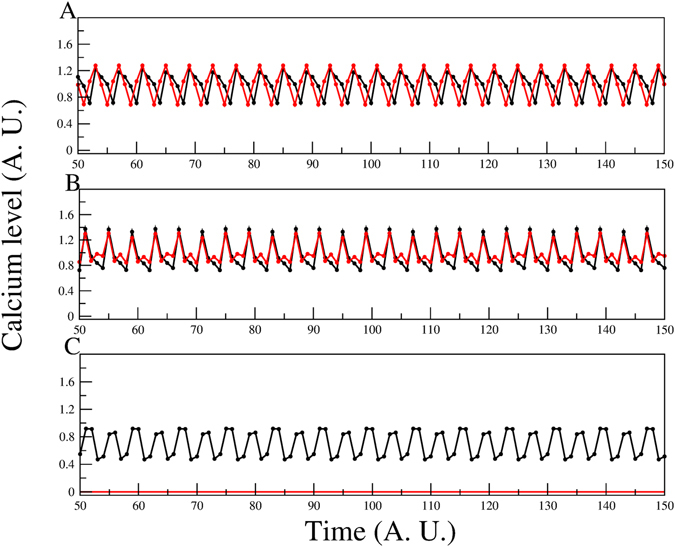
[*Ca* ^2+^]_*i*_ dynamics of the network deleting the pH node for the three models. In panel A for Model-I calcium dynamics, with all nodes present (black) and without the pH node (red), notice that there is hardly any difference in the level of calcium between the two situations. A similar situation is present in panel B for Model-II. The case of Model-III is shown in panel C, the calcium fluctuations completely disappear in the absence of pH, this is so, because the pH directly controls the CatSper dynamics.

[*Ca* ^2+^]_*i*_ oscillations are expected to be pH_*i*_ sensitive^31^. In Fig. 5, for Model-I (panel A), though the effect of pH_*i*_ variations is indirectly modulated via a cAMPCC, the steady state oscillations in presence of HVA and LVA are basically unperturbed by the pH blockage. A similar situation is registered in panel B, for Model-II, now by the effect of CatSper instead of cAMPCC. For Model-III the direct connection to CatSper, in the absence of LVA and HVA causes a collapse in *Ca* ^2+^ levels if the pH node is deleted. This is shown in panel C.

### Deletion of the CaKC Channel resolves a previous model prediction shortcoming

**Figure 6:**
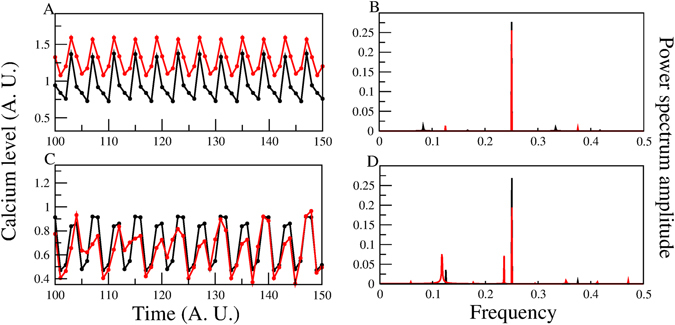
[*Ca* ^2+^]_*i*_ dynamics of the network model with and without the CaKC channel for Models II and III. In panel A for Model-II the [*Ca* ^2+^]_*i*_ node dynamics with all nodes present is shown in black and without the CaKC channel in red. Notice the higher calcium concentration in the red curve. A longer period for the WT is manifest in panel B by the appearance of an additional lower frequency Fourier mode. For panels (C) and (D), the Model-III calcium dynamics shows a decrease in the calcium level (as observed in the experiments with Iberiotoxin)^16^ as well as a more elaborate temporal behaviour in the Fourier power spectrum with the appearance of a longer period component not present in the WT case (also observed in the mentioned experiment).

In ref. 16, from visual inspection of the evolution of individual initial conditions we predicted with Model-I network, that deletion of the CaKC channel produces an increase in the interval between successive [*Ca* ^2+^]_*i*_ oscillations, as well as an elevation on the average [*Ca* ^2+^]_*i*_. This prompted *S. purpuratus* experiments, also reported in ref. 16, on the effect of using Iberiotoxin, a powerful and specific blocker of the *Ca* ^2+^-dependent *K* ^+^ channel Slo1, which showed an increase of the interval between successive *Ca* ^2+^ oscillations, hence corroborating the prediction. However, a qualitative perception of the fluorescent intensity of the [*Ca* ^2+^]_*i*_ fluctuations was indicative of a lower average value than the WT case. Here, by considering the *Ca* ^2+^ now averaged over 100,000 initial conditions we note in panel A of Fig. 6 that for Model-II, *Ca* ^2+^ continues to be higher than the WT (in disagreement with the experiment) and the periodicity apparently is not substantially modified (again in contrast to experiment). To quantify the temporal behaviour of the *Ca* ^2+^ dynamics, we performed a Fourier Spectrum Analysis.

In a nutshell, peaks of the power spectra are indicative of the most important frequencies in these time series (Fourier modes). Since we have chosen the frequency representation in our Fourier analysis, the inverse of the frequency associated to each Fourier mode, corresponds to the most important periods of the calcium dynamics. These modes are dependent of the periodicity of the steady states reached by the whole network dynamics.

Our Fourier Spectrum analysis, performed as in ref. 34, indicates the presence of a lower frequency mode in the WT, i.e. there is a larger period component in the WT with regard to the CaKC blocked case (Fig. 6B). This, in contrast with the behaviour of Model-III, shown in panels C and D, where both experimental observations are retrieved: namely an increase in the period (reflected by the more elaborated Power Spectrum) and a decrease in the average *Ca* ^2+^ concentration.

### CatSper channel explains experiments of the effect of niflumic acid on *Ca*^2+^ transient behaviour

**Figure 7:**
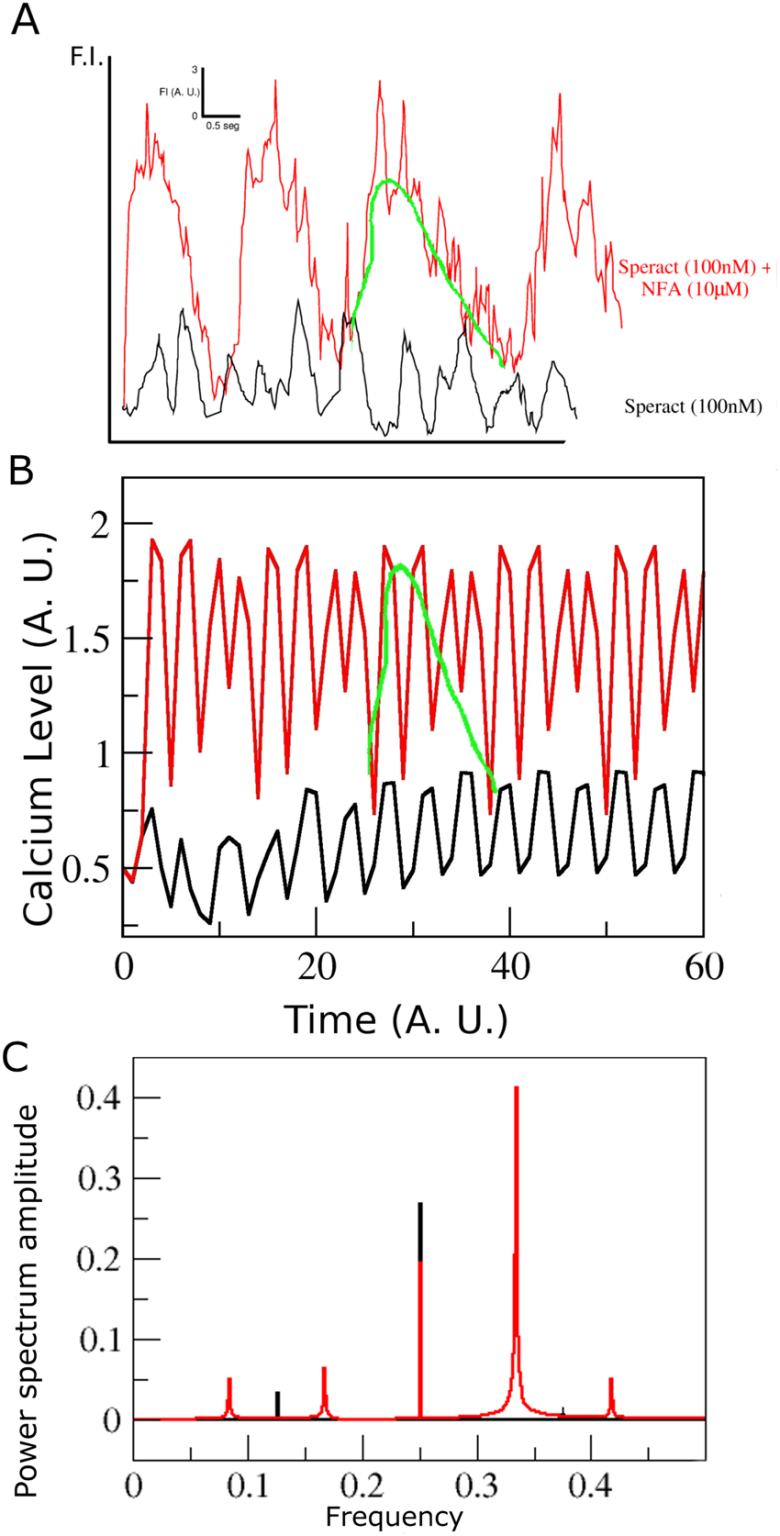
Comparison between experiments on the effect of niflumic acid (NFA) and the network dynamics of Model-III. (**A**) Experimental [*Ca* ^2+^]_*i*_ curves using 100 nM of speract (black) and 100 nM of speract + 10 *μ*M of NFA (red). Notice that the average [*Ca* ^2+^]_*i*_ mean, amplitude, maximum peak and interval between peaks is higher in the red curve (with NFA) than in the WT curve. (**B**) *Ca* ^2+^ time series generated from Model-III with all nodes present in black, and in red the “NFA case”, with HCN, CaCC channels blocked and CaKC, CatSper channels over activated. The green curve is placed as a guide to the eye for an envelope of both experiment and model series. (**C**) Fourier spectra of the above time series, with same colour code. Notice the more elaborate temporal behaviour and higher period component in the red curve.

In our previous work^34^, a study in terms of Model-I of the action of Niflumic Acid (NFA) on the flagellum membrane of *S. purpuratus* pointed either to the need of an additional *Ca* ^2+^ channel such as the pH-dependent CatSper channel, not taken previously into account in its signaling pathway, or to the participation of a CaKC channel different from Slo1. With either choice, it was expected that the experimental determinations carried out in ref. 17 for the case of homogeneously distributed speract could be attained *in silico*, namely: an increase in the average *Ca* ^2+^ intracellular concentration, fluctuation amplitude, peak values and in the interval between successive oscillations. With Model-I, under the assumption that NFA blocks the HCN and CaCC channels and activates the CaKC channel, the experimental results are attained except for the latter one. In Fig. 7, with the suggested inclusion of CatSper in Model-III, we show that all the experimental observations are recovered: Fig. 7A shows a modified version of the experimental results^17^. Figure 7B shows that amplitude, peak and average values clearly recover experiment. The green line is a guide to the eye for the structures behind the higher periods in experiment and simulation. Figure 7C is the Fourier spectrum of the *Ca* ^2+^ fluctuations averaged out over 100,000 initial conditions resulting from the dynamics of Model-III. Notice the presence of Fourier modes with periods higher than those encountered in the WT (i.e. in absence of NFA), coincident with the experimental determinations (red peaks at the left side of the figure).

This assertion is reinforced by the joint analysis of the model-generated [*Ca* ^2+^]_*i*_ fluctuations and their Fourier transforms (panels C and B of Fig. 7, respectively). The Fourier power spectrum modes of Model-III, identified by the peaks in Fig. 7, show the relative weight of periodic components in the [*Ca* ^2+^]_*i*_ signal in the presence (red) and absence (black) of NFA. The first red peak to the left corresponds to a period-12 Fourier mode, whilst the first black Fourier mode to the left of the wild type is indicative of a period-8 contribution. The link between the 12 steps red oscillation structure encompassed by the green line, that repeats itself periodically after 12 iterations, together with the period 12 Fourier mode, provides a manifestation of period elongation that goes beyond a guide to the eye. In general, Fourier analysis provides a means for exploring underlying regular components in the *Ca* ^2+^ series, such as the coexistence of several initial condition dependent steady states. A more detailed study of the effect of NFA on the *Ca* ^2+^ fluctuations, based on Fourier spectra can be found in ref. 34.

If the same calculations are carried out for Model-II, with the inclusion of the HVA and LVA channels, we obtain Supplementary Fig. S1. In this case the increase in average *Ca* ^2+^ and average peak values is evident. However, contrary to the experimental results, the interval between successive oscillations and the fluctuation amplitudes are diminished.

### Experimental validation

#### Mibefradil and NNC, two known CatSper inhibitors, affect the speract induced [*Ca*^2+^]_*i*_ increase in *S. purpuratus* sea urchin sperm

**Figure 8:**
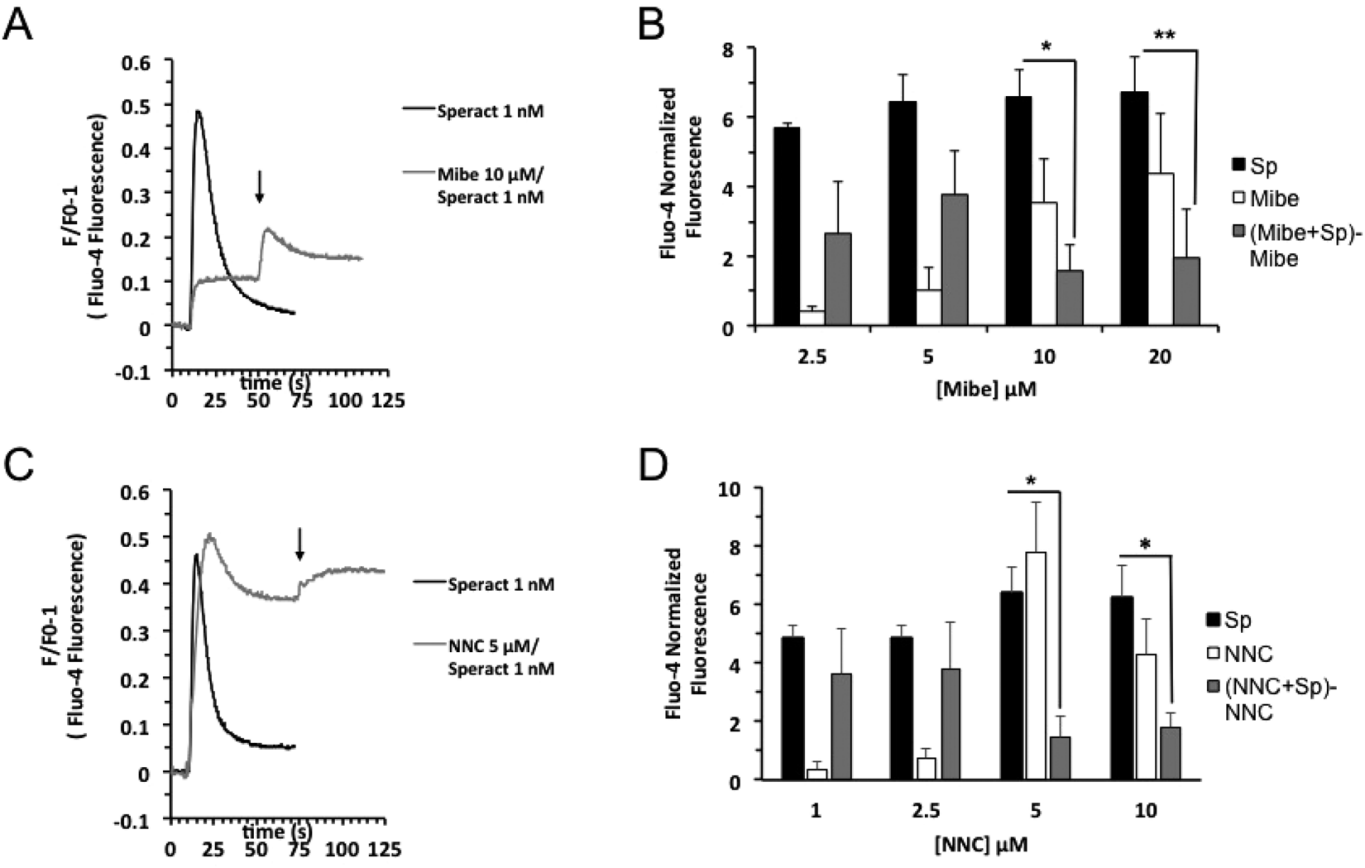
Catsper participates in the speract signaling cascade. Fluo-4 labelled *S. purpuratus* sea urchin sperm diluted (1:10) in 1CaSW pH 7.0, were further diluted (5 *μ*l) in 800 *μ*l ASW pH 7.8. After recording the fluorescence for 10 seconds, 1 nM speract (**A** and **C**), 10 *μ*M Mibefradil (Mibe) (**A**), or 5 *μ*M NNC-055-396 (NNC) (**C**), were added. Channel blockers were incubated for 1 min. In (**A** and **C**) arrows indicate speract additions. (**B** and **D**) Summary of experiments performed as in (**A**) or (**C**) respectively, assessing the fluorescence change in the maximum after additions. The Fluo-4 normalized fluorescence (%) was obtained considering the fluorescence obtained after adding 0.05% Triton-X100 as 100%. Bars represent the mean ± s.e.m. (**B**) *n* = 3–8. (**D**) *n* = 4–5. *p < 0.001 and **p < 0.02.

The results obtained by the model network simulations motivated us to devise pharmacological experiments and examine the participation of CatSper channels in the SASP. Mibefradil and NNC-055-396 (NNC), two CatSper inhibitors^46, 47^, were tested in the speract-induced [*Ca* ^2+^]_*i*_ increase, though both also block T-type voltage-dependent *Ca* ^2+^ channels (LVA)^50, 51^ (see discussion). These compounds were shown to inhibit the progesterone induced [*Ca* ^2+^]_*i*_ increases mediated by CatSper in human sperm^52^. Furthermore, during the course of these experiments, Seifert and colleagues^32^, showed all CatSper subunits are expressed in *A. punctulata* sperm and mibefradil was used to identify the participation of CatSper in chemotaxis. Figure 8 illustrates that both Mibefradil and NNC significantly, but no completely, inhibit the speract induced elevation of [*Ca* ^2+^]_*i*_. These findings support the contention that CatSper channels have an important role in the response to speract and therefore in chemotaxis.

#### Most CatSper subunits are present in *S. Purpuratus* sperm

We attempted to corroborate by proteomics the presence of voltage-dependent *Ca* ^2+^ Cav channels we had detected by immunocytochemistry in the flagella of sea urchin sperm^8^ and explore CatSper protein expression. Isolated flagella membranes^53^ from *S. purpuratus* were extracted with Triton X-114 to obtain a fraction enriched with integral membrane proteins^54^. The proteins in the Triton X-114 phase were resolved in either 10% (Supplementary Fig. S2A–C) or 7.5% (Supplementary Fig. S2D and E) polyacrylamide electrophoresis mini gels under denaturing conditions (SDS-PAGE). The gel was either stained with GelCode Blue Safe (#1860957 Thermo Scientific) or used to detect Cav channels by western blot (WB) experiments using Anti-Cav-PAN (#ACC-004; 1:200) and Anti-Cav1.2 (#ACC-003; 1:200) from Alomone Labs, raised against mammalian proteins. Two main bands were detected with Anti-Cav-PAN (Supplementary Fig. S2B) and Anti-Cav1.2 (Supplementary Fig. S2C), and the corresponding areas were excised from the gel (Supplementary Fig. S2A) and sent to the Proteomics Facility at the Institut de Recherches Cliniques de Montreal, Canada to be sequenced by LC-MS/MS as described^55^. Against expectations, the presence of different unique peptides for three CatSper family members (Catsper 1, 2 and 4) and the *β*, *δ* and *γ* subunits of this channel (Table 1) were identified among other proteins, as reported for *Arbacia punctulata* ^32^. To validate the presence of the CatSper channel in the Triton X-114-extracted flagella membranes, we used an Anti-CatSper1 antibody raised against two peptides (BSYN 6765 and BSYN 6766; Bio-Synthesis, Inc) based on the *S. purpuratus* CatSper1 predicted sequence (XP_011664201). The stained SDS-PAGE (Supplementary Fig. S2D), was divided in 12, 0.5 mm sections and sequenced by LC-MS/MS. Supplementary Fig. S2E shows the WB of the 1st (line 1) and 2nd (line 2) Triton X-114-extracted flagella membranes labelled with Anti-CatSper1 (1:1000). Although the calculated MW for CatSper1 is 54440 Da, we did not observe it under these conditions in which CatSper1 forms different aggregates. Catsper beta and gamma were identified in the area indicated by “*” (sections 5 & 6) in the stained SDS-PAGE. None of the bands analyzed by LC-MS/MS contained Cav channels suggesting that they are in very low representation in the flagella membranes comparing with the CatSper channel, or absent. Additionally, we identified by tandem mass spectrometry exclusive unique peptide sequences for a calcium-activated potassium channel in *S. purpuratus* sea urchin sperm flagellum (NCBI Reference Sequence: XP_011666091.1 (GI:115621189); MW: 144 kDa) (Supplementary Table S1).

## Discussion

The relevance of intracellular *Ca* ^2+^ fluctuations triggered by SAPS to sea urchin swimming has long been established^1, 56^, however the identification of the flagellum *Ca* ^2+^ channels that contribute to these transients is a matter of current research^24, 32^. Though the presence and participation of the CatSper channel in *A. punctulata* sperm chemotaxis was recently shown^32^, the degree of its contribution has not been determined, leaving open the question of the participation of other *Ca* ^2+^ channels such as the LVA and HVA to this process. In this paper we address this issue by means of a discrete network model for the dynamics of the *Ca* ^2+^ signaling pathway for *S. purpuratus*, triggered by speract, which has previously contributed to the understanding and prediction of some properties of the *Ca* ^2+^ transients^16^. In particular, the need of a channel such as CatSper was predicted by this model in order to retrieve experimental results related to the action of the NFA drug^34^. Here, the exploration of three model variants characterized by the *Ca* ^2+^ channels being considered: Model-I with HVA and LVA, Model-II with CatSper, LVA and HVA, and Model-III only with CatSper, has enabled us to test the contribution of these channels and to gauge their relative importance guided by criteria based on experimental corroboration. Our systems biology approach allowed us to consider the behaviour of the full network dynamics and the effect of the action of several drugs. Our findings are summarized in Table 2, where the comparison of four experimental determinations (sensitivity to pH_*i*_ levels, sensitivity to PDE concentration, CaKC blockage and action of NFA) with the model determinations is shown.

**Table 2:** Comparative model-experiment study of the three network models. First column indicates the pharmacological treatment under consideration; column 2 specifies the quantity being measured; column 3 are Model-I results; column 4 Model-II results; column 5, Model-III results; column 6, experimental determinations. ↑ correspond to an increase in the calcium property indicated in the measurement column. ↓ is associated with a decrease and “−” stands for minor changes. The matching index is defined as the ratio between the number of model/experiment coincidences and the total number of experimental determinations. A matching index of 1 indicates full agreement between experiment and specified model results.

Treatment	Measurement	Model-I	Model-II	Model-III	Experiment
pH blockage	Ca level	—	—	↓	↓
PDE blockage	Ca level	—	—	↓	↓
CaKC blockage	Ca level	↑	↑	↓	↓
	Period	↑	↓	↑	↑
NFA addition	Ca level	—	↑	↑	↑
	Peak	↓	↑	↑	↑
	Amplitude	↓	↓	↑	↑
	Period	↓	↓	↑	↑
	Matching Index	0.25	0.375	1	

The table clearly indicates that best results are obtained with Model-III and that Model-II has some advantages over the Model-I. The detailed interpretations of this findings can be consulted in the results section. Our model hence predicts that assuming a 1-to-1 stoichiometry of all network elements, CatSper is indispensable and appears to be the main *Ca* ^2+^ channel. It is worth noting that CatSper is present in the *S. purpuratus* genome^57^ and that here we corroborate that most of its reported protein subunits are expressed in sperm, as determined by proteomics. As anticipated, two CatSper blockers inhibit the speract induced [*Ca* ^2+^]_*i*_ increase, however this inhibition was partial. These results could suggest that another *Ca* ^2+^ transport system participates. Recent observations in single *S. purpuratus* sperm showed that the speract-induced increase in flagellar *pH*_*i*_ precedes and is independent of that of [*Ca* ^2+^]_*i*_, therefore this alkalinization could directly activate a *Ca* ^2+^ channel such as CatSper. However, an alkalinization induced by *NH*_4_ *Cl*, which is larger than that caused by speract and that produces a membrane potential depolarization^31^, results in less *Ca* ^2+^ influx, as compared with that triggered by speract. This finding would also be consistent with the required participation of another *Ca* ^2+^ uptake mechanism in the speract response^24^.

Given the lack of specificity of CatSper blockers: NNC and mibefradil, neither our pharmacological experiments nor those presented in ref. 32 prove that CatSper is key to sea urchin sperm chemotaxis. Under these circumstances the revelation of our network dynamics of the dominance of CatSper in the regulation of [*Ca* ^2+^]_*i*_ oscillations, triggered by speract comes as an important theoretical prediction which requires alternate experimental confirmation. Besides the analysis here reported, credence of this result is further supported by previous successes of this approach in corroborating and predicting experiments^16, 34^. A predictive capacity in the modeling of fundamental biological phenomena is encouraging and seldom encountered. In this respect a Systems biology approach with strong experimental backing is promising.

The stoichiometry mentioned above is an important consideration. Additionally, it may be that a model in which the participation of the diverse channels is properly weighted may render a Model-II type network more appropriate. Further experimental work and alternative theoretical formulations such as continuum dynamics studies are required for a more thorough and conclusive analysis.

With regard to the other *Ca* ^2+^ channels, in Fig. 2 we observe that deletion of Cavs modifies the *Ca* ^2+^ curve with a faster decay, thus influencing the dynamical behaviour of the system under that perturbation, but maintaining almost unaltered the average calcium concentration as well as the elevation rate. Since phenomena like chemotaxis and chemokinesis are strongly related to the temporal synchronization of the signaling pathway and the molecular machinery with spatio-temporal gradients of egg-derived molecules^2, 15^, we suggest that the the presence of those channels could intervene in the fine tunning necessary for a correct movement of the sperm in its searching for the egg.

## Materials and Methods

### Construction of the speract-activated network model

The construction of the regulatory functions mentioned in the SASP network model section is better understood by means of an example regarding the regulation of cyclic GMP (cGMP). This molecule is a well known second messenger in almost every cell type. It is synthesized by guanylate cyclase (GC) and degraded by the phosphodiesterases (PDE). In this case, cGMP, GC and PDE take two values: 0 and 1. For the construction of its regulatory function it is necessary to take into account all possible combinations of nodes linked to it. At time t, if PDE is active (state 1), cGMP cannot be synthesized, even if GC is active. This is because PDE is a strong inhibitor. When PDE state is 0, if GC or cGMP are in state 1, cGMP will be on at time (t + 1). This table has positive autoregulation of cGMP. Table 3 shows the above process by means of the regulatory function (or truth table) which relates cGMP with its 8 regulatory combinations.

**Table 3:** cGMP regulatory table.

GC (*t*)	PDE (*t*)	cGMP (*t*)	*cGMP*(*t* + 1)
0	0	0	0
0	0	1	1
0	1	0	0
0	1	1	0
1	0	0	1
1	0	1	1
1	1	0	0
1	1	1	0

The first 3 columns in this table contain all the possible activation states of the cGMP regulators (GC, PDE, cGMP); the fourth column shows the values for the function that correspond to each combination of those regulators.

This construction was applied to all nodes in the system. It is important to mention that several criteria can intervene in the construction of a regulatory function and in order for it to be meaningful an in depth biological knowledge is essential.

### Elimination and activation of nodes during the network dynamics

For node elimination in the network dynamics, a zero value was imposed on it during the whole dynamics independently of its regulators. In the particular case of CaKC and CatSper, though there are evidences in mouse sperm suggesting CatSper is stimulated by low NFA concentration^34^, we have assumed this occurs in sea urchin sperm. We simulated an activation effect by forcing them to take the value of 1 during a certain period, for example 3 steps, and then releasing them in the fourth step. Actually, the network dynamics results are not strongly dependent on the specific values of the above steps. For the Fig. 7, we forced the CaKC and CatSper channels to a value of 1 during three of four steps.

### [*Ca*^2+^]_*i*_ dynamics time series

We determined model generated speract-triggered [*Ca* ^2+^]_*i*_ fluctuations by averaging the value of the [*Ca* ^2+^] node for each step in the dynamics over 10^5^ different initial conditions of the entire network (with all nodes present) once steady state periodic oscillations were reached. Since our nodes take a finite number of values and the network is finite, periodic behaviours are always attained. Though the initial conditions are chosen according to a uniform distribution, oscillatory phases are determined by the distribution of the time of arrival to the periodic steady state. In our study we compared averaged intracellular *Ca* ^2+^ ion concentration fluctuations determined from the model dynamics under consideration with experimentally measured [*Ca* ^2+^]_*i*_ levels determined as averaged values taken over flagellum length. With this procedure we obtain a finer resolution for the *Ca* ^2+^ concentration, more adequate for comparison with experimental measurements, as well as more appropriate for the calculation of systemic features.

Starting out the dynamics at time *t* = 0 from any given initial state, the network will traverse through a series of transitory states until it reaches a periodic pattern of activity called *attractor*. All the initial conditions that end up in a given attractor constitute the *basin of attraction* of that attractor. Several attractors may coexist for the same network, each one with its own basin of attraction. The Fourier power spectrum modes are dependent of both, the period of the attractors and also their basins of attraction.

### Evaluation of CatSper robustness

In order to assess the stability of our network model including the CatSper channel, we performed a robustness analysis. Our robustness test consists of changing in the regulatory rule for the CatSper node, the outcome, one row at a time. This is a structural stability test. For every change in the regulatory function of CatSper, a *Ca* ^2+^ time series is obtained after averaging over 100,000 initial conditions. A Pearson correlation coefficient was determined between all pair of time series consisting of the wild type (WT) and each of the 18 different modifications to the regulatory rule. Highly correlated *Ca* ^2+^ series have a correlation coefficient close to 1, while an uncorrelated behaviour leads to values close to 0. It is worthwhile to mention that for both cases, model-II and model-III, the Pearson correlation coefficients are close to 1, indicating that the systems are robust to most of the single row perturbations (Fig. S3). Low correlation values correspond to highly unlikely behaviours, such as the case of switching the CatSper channel state from close to open during a hyperpolarization and basal pH (values = 0).

### Sea urchin and sperm collection

*Strongylocentrotus purpuratus* sea urchins were obtained from Pamanes S.A. de C.V. (Ensenada, Baja California, México). Sperm were obtained by intracoelomic injection of the sea urchins with 0.5 M KCl, collected and kept on ice as dry sperm until used (within one day). Artificial seawater (ASW) contained (in mM): 485 NaCl, 10 KCl, 10 CaCl_2_, 26 MgCl_2_, 30 MgSO_4_, 2.5 NaHCO_3_, 10 Hepes, 0.1 EDTA, pH 7.8. The 1CaASW is ASW pH 7.0, containing 1 mM CaCl_2_. In both cases the osmolarity was 950–1000 mOsm. NNC 55–0396 was from Tocris Bioscience (Minneapolis, MN), the GelCodeTM Blue Safe Protein Stain (24594) was from Thermo Scientific. Fluo-4-AM was obtained from Molecular Probes, Inc. (Eugene, OR, USA), and Mibefradil and the rest of the reagents were from Sigma-Aldrich.

### Sample preparation and protein identification by tandem Mass Spectrometry (LC-MS/MS)

Membranes from *S. purpuratus* sea urchin sperm flagella were isolated^53^, and extracted with Triton X-114^54^, as described. Protein bands from a 10% SDS-PAGE stained with GelCode Blue Safe were cut in 4 mm high and 10 mm width fragments and sent to the Proteomics Facility at the Institut de Recherches Cliniques de Montreal, Canada, and process as previously described^55^. All MS/MS samples were analyzed using Mascot (Matrix Science, London, UK, version 2.5.1). Mascot was set up to search the NCBI_ S_Purpuratus_20111201 database assuming the digestion enzyme trypsin. Mascot was searched with a fragment ion mass tolerance of 0.52 Da and a parent ion tolerance of 10.0 PPM. *O* + 18 of pyrrolysine and carbamidomethyl of cysteine were specified in Mascot as fixed modifications. Oxidation of methionine was specified in Mascot as a variable modification. Scaffold (version Scaffold_4.2.1, Proteome Software Inc., Portland, OR) was used to validate MS/MS based peptide and protein identifications. Peptide identifications were accepted if they could be established at greater than 95.0% probability by the Peptide Prophet algorithm^58^ with Scaffold delta-mass correction. Protein identifications were accepted if they could be established at greater than 99.0% probability. Protein probabilities were assigned by the Protein Prophet algorithm^59^.

### Fluorometric determinations of [*Ca*^2+^]_*i*_ in sperm populations

Sperm fluorescence measurements were done as described^60^ with a 5 *μ*l aliquot of Fluo-4 labelled sperm (diluted 1:5 in 1CaASW pH 7.0) added to a round cuvette containing 800 *μ*l ASW at 14 °C under constant stirring in a SLM 8000 Aminco spectrofluorometer. Fluo-4 has been used to detect speract induced [*Ca* ^2+^]_*i*_ oscillations in individual sea urchin sperm and in populations. Controls have been done showing that these fluctuations are not due to Fluo-4 pH-dependent fluorescent changes.

#### Statistics

Fluorescence records were acquired with 4.2 Global Works OLIS software and processed with Microsoft Excel. The values given in the bar plots are the mean ± s.e.m and n = independent experiments. For statistical evaluations we used the Student’s t-test. Differences were considered statistically significant when *p* < 0.05.

## Electronic supplementary material

Supplementary Information

Dataset S1

Dataset S2

Dataset S3
