# Supplementary material for: Network model predicts that CatSper is the main Ca2+ channel in the regulation of sea urchin sperm motility
Source: Sci Rep. 2017 Jun 26;7:4236. doi: 10.1038/s41598-017-03857-9 (PMC5484689; doi:10.1038/s41598-017-03857-9)
Supplement: Supplementary file 1 — Supplementary Information [file 41598_2017_3857_MOESM1_ESM.pdf]

Network model predicts that CatSper is the main Ca^{2+} channel in the regulation of sea urchin sperm motility

Jesús Espinal-Enríquez, Daniel Alejandro Priego-Espinosa, Alberto Darszon, Carmen Beltrán and Gustavo Martínez-Mekler.

Supplementary material

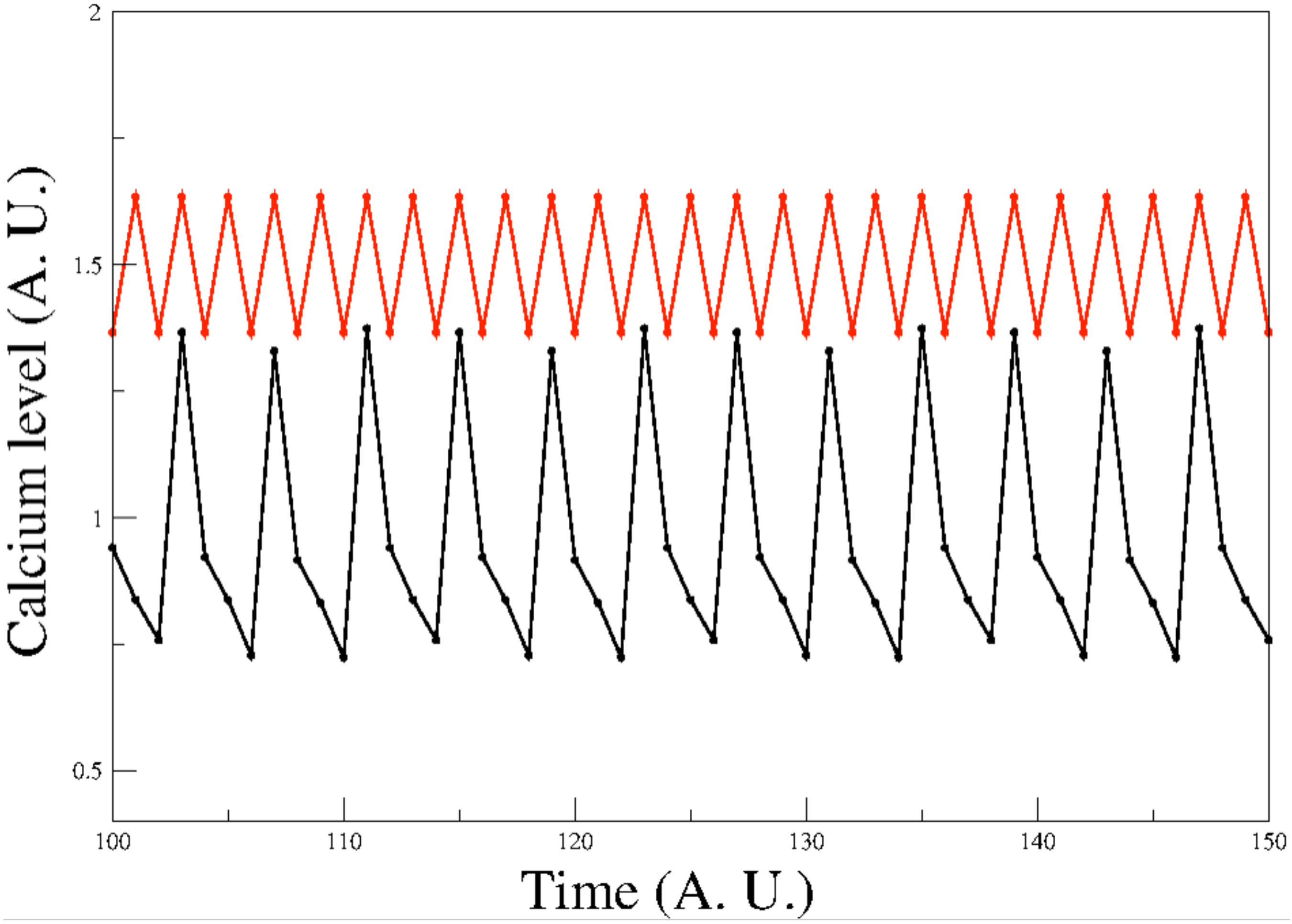

Figure S1
In silico effect of NFA in Model-II Ca^{2+} time series. The calculation was performed as in Fig. 2. Black line represents the WT case, meanwhile the red one indicates the effect of NFA: activation of CaKC and CatSper channels, and inhibition of CaCC and HCN channels. Notice the increase in the average and maximum peak in the red curve compared to the black one, as well as the reduced periodic behavior and amplitude with respect to the WT.

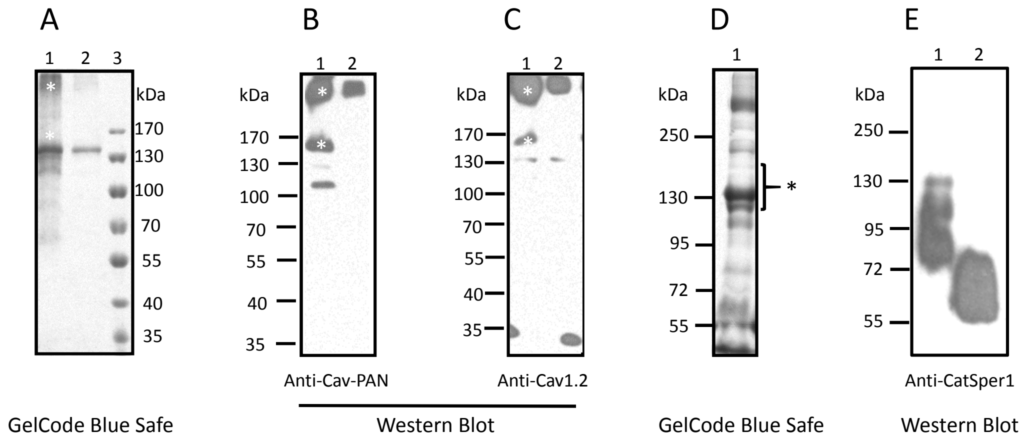

Figure S2

Cav and CatSper1 channels are present in Triton X-114-extracted flagella membranes from *S. purpuratus* sperm. A) and D) are GelCode Blue Safe stained gels. B), C) and E) are western blots (WB) of the proteins revealed with Anti-Cav-PAN (B), Anti-Cav1.2 (C) and Anti-Catsper (E) respectively. In A)-E), lines 1 and 2 contain the proteins from the 1st and 2nd Triton X-114-extracted flagella membranes respectively. Line 3 are the MW standards PageRulerTM Plus Pertained Protein Ladder from Fermentas. In A) and D), ``*'' indicate the gel areas that were excised for LC-MS/M analysis. Please refer to the section: ``Most CatSper subunits are present in *S. purpuratus* sperm'', for a detailed description.

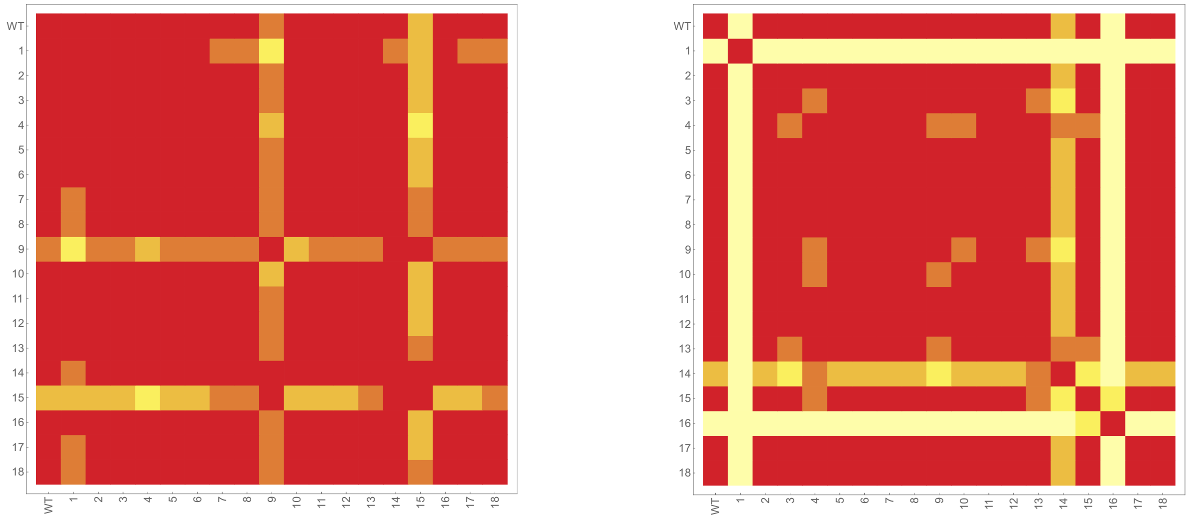

Figure S3: Correlation matrices for model II (left) and III (right) showing the effect of perturbation of the regulatory rule of CatSper channel. The axes indicate the outcome row of the regulatory function that has been altered. Each square represents the Pearson correlation between 1,000 time steps calcium dynamics after the steady state is reached. Red squares correspond to high correlations (close to 1), meanwhile the yellow ones are low correlation values (close to 0). Notice that most of the area of both matrices is covered by red squares.

Table S1 Amino acid peptide sequences of the calcium-activated potassium channel subunit alpha-1 (CaKC) [*S. purpuratus*]. Peptide sequences were obtained by tandem mass spectrometry (LC-MS/MS). *AffCol: Affinity Column; Cobalt: Co^{2+}; **SDS-PAGE 10% /Western Blot (Anti-Cav-PAN: ACC-004, Alomone Labs Ltd); WGA: Wheat germ agglutinin. Flagela and Flagellar Membranes were obtained as in Darszon *et al.*, 1994

	**Table S1 Amino acid peptide sequences of the calcium-activated potassium channel subunit alpha-1 (CaKC) [*S. purpuratus*]** (NCBI Reference Sequence: XP_011666091.1 (GI:115621189); MW: 144 kDa)			
File Sequencing		**Exclusive Unique Peptide Sequence**	**Sperm sample**	**Purification Method**
20130108	1	EYSASLISAQTKI	Flagella	AffCol_WGA pH 8*
20140221	2	EYSASLISAQTK	Flagella	Band I **
20140221	3	EYSASLISAQT	Flagella	AffCol_Co^{2+}
20130108	4	SLEEATIDR	Flagella	AffCol_WGA pH 8*
20130108	5	TIVLLGNIDYIK	Flagella	"
20130108 (2)	6	NYGILCFGIYR	Flagella	"
20140221 (2)	7	YSGSLQSEK	Flagella	"
20140221	8	HIVVCG	Flagella	"
20140221 (2)	9	HFTQLQYFQGSVLNSVDLER	Flagella	AffCol_WGA pH 3*
20140221	10	TLITGGATPELEQILAEGGGMKPGQNNVELLANR	Flagella	"
20150420	11	SLEEATIDRTQAHK	Flagellar Membranes/TX-114 extracted	Band I **
20150420	12	GKYSGSLQSEK	Flagellar Membranes/TX-114 extracted	"
20150420	13	TGGCPRPSVGSLGQLVLKL	Flagellar Membranes/TX-114 extracted	"
20150420	14	VFVLNGSPLSR	Flagellar Membranes/TX-114 extracted	"
20150420	15	FRDSSASTSTPSSK	Flagellar Membranes/TX-114 extracted	"
20150420 (2)	16	TVKEQPEKEKPSER	Flagellar Membranes/TX-114 extracted	Band I/II **

Dataset S1

Model-I regulatory rules. The construction of the three network models was performed as explained in the Methods section. These Model-I regulatory rules have been published previously in: Espinal J, Aldana M, Guerrero A, Wood C, Darszon A, Martínez-Mekler G (2011) Discrete Dynamics Model for the Speract-Activated Ca^{2+} Signaling Network Relevant to Sperm Motility. PLoS ONE 6(8): e22619. doi:10.1371/journal.pone.0022619

Dataset S2

Model-II regulatory rules.

Dataset S3

Model-III regulatory rules.
